# A Case of Cerebral Vasculitis Associated with Ulcerative Colitis

**DOI:** 10.1155/2015/598273

**Published:** 2015-10-18

**Authors:** Naveen Raj, Matthew Arkebauer, Barry Waters, Brucha Dickinson

**Affiliations:** ^1^Department of Rheumatology, Nova Southeastern University, Larkin Community Hospital, Miami, FL, USA; ^2^Nova Southeastern University, Larkin Community Hospital, USA; ^3^Arthritis Specialists PA Coral Springs, FL, USA; ^4^Department of Pathology, Florida Medical Center, Fort Lauderdale, FL, USA

## Abstract

Ulcerative colitis (UC) is a chronic, debilitating condition characterized by inflammation of the colonic mucosa. It is regarded as a systemic inflammatory disorder that can affect a number of organ systems. Central nervous system disease associated with UC is a rare sequela of inflammatory bowel disease, occurring in less than 5% of cases. These manifestations include arterial and venous thrombosis, leukoencephalitis, seizures, and vasculitis. We present a case of a 61-year-old female with a two-year history of well-controlled ulcerative colitis, who developed altered mental status and weakness. On brain imaging, she was found to have cerebral lesions which were biopsied. Histopathology subsequently revealed coagulative necrosis and inflammation characteristic of vasculitis. Rheumatology serologies were negative, and the patient was started on steroids that dramatically improved her neurological function, with no residual deficits, and led to resolution of the brain lesions.

## 1. Introduction

UC and Crohn's disease (CD) are both chronic, relapsing inflammatory bowel disorders (IBD) of unknown etiology, in which disturbed immunologic processes are thought to play a role in their pathogenesis [1]. IBD is considered a systemic illness, as many organs outside the gastrointestinal tract can be affected. Indeed, in a 2005 editorial in* Gastroenterology*, it was noted that “the effects of inflammatory bowel disease (IBD) extend to every corner of the body” [2, 3]. Extraintestinal manifestations include ocular (uveitis and iritis), hepatic (primary sclerosing cholangitis and autoimmune hepatitis), joints (arthritis and ankylosing spondylitis), bones (osteoporosis), blood (anemia, hemolytic anemia, myelodysplastic syndrome, and thrombosis), lung (asthma and bronchiolitis obliterans), and skin (pyoderma gangrenosum, erythema nodosum, and aphthous stomatitis) [1, 2]. These manifestations collectively can occur in up to 25% of UC patients in their lifetime [4]. However, central nervous system (CNS) manifestations of UC are quite rare, and estimates are hard to come by. In a retrospective register-based study of 638 patients with UC or CD, Lossos et al. found neurological involvement in 3% of the cases [1, 5].

## 2. Case Summary

A 61-year-old Caucasian female was admitted to the hospital with acute altered mental status (AMS). On arrival to the emergency room, her vital signs were T: 98.6 F, P: 67, BP: 190/78, RR: 20, and 100% O_2_ saturation on room air. On examination she appeared disheveled, lethargic, but arousable to loud voice. She was unable to recite her address or state what year it was. She had a normal rate and rhythm with no murmurs on heart exam. Lungs revealed diminished breath sounds, but no rhonchi or wheezing. Abdomen was soft, nontender, and nondistended. She had no pedal edema. Skin revealed no rashes or trauma. On neurological evaluation, she was oriented to person only. She did follow some basic commands. Cranial nerve exam revealed no facial asymmetries, and tongue protrusion was midline with no bite marks. She had 2+ patellar and ankle deep tendon reflexes, with a negative Babinski reflex. No clonus was appreciated. Pupils were reactive and anicteric. On motor exam, she exhibited a poor effort but did move both upper extremities and the left lower extremity minimally. There was no movement in the right lower extremity. Sensory and cerebellar test were unobtainable due to her AMS. Pulses were normal in bilateral upper and lower extremities.

**Figure 1:**
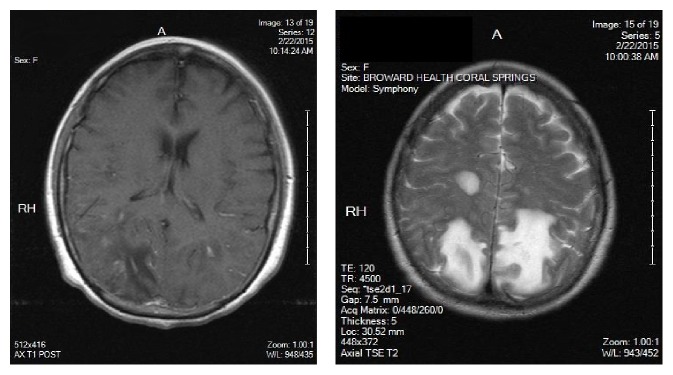
Brain MRI on admission: multiple enhancing masses in the bilateral occipital and posterior parietal lobes on T1 and T2. Enhancement in the right centrum semiovale is also visualized.

Her past medical history included hypertension and UC. She was diagnosed with UC two years ago via colonoscopy and bowel biopsy. At that time, her symptoms were abdominal pain and bloody diarrhea. She was placed on mesalamine 800 mg by mouth twice a day, which controlled her symptoms, and did not require any escalation or change in treatment. In addition to mesalamine, her other current medications were amlodipine 5 mg daily and benazepril 10 mg daily for hypertension and ferrous sulfate 325 mg daily for iron deficient anemia. She did not have any surgical history, and no known drug allergies were observed. She did not smoke or partake in illicit drugs. Initial pertinent labs are in Table 1(a). She had a normal sinus rhythm by EKG, and chest X-ray showed no abnormalities. A computer tomography scan (CT) of her brain showed bilateral deep white matter and subcortical hypodensities in the parietal and occipital lobes concerning for infarction. A neurologist was consulted, and a brain magnetic resonance image (MRI) with and without contrast was ordered, which showed large areas of multiple enhancing masses predominantly involving the bilateral occipital and posterior parietal lobes (Figure 1). There was also a rounded focus of edema in the central right parietal lobe at the centrum semiovale. She was started on anticoagulation with full dose aspirin. Her blood pressure medications were continued. The differential diagnosis at this point included infection, malignancy, and Posterior Reversible Encephalopathy Syndrome (PRES).

**Figure 2:**
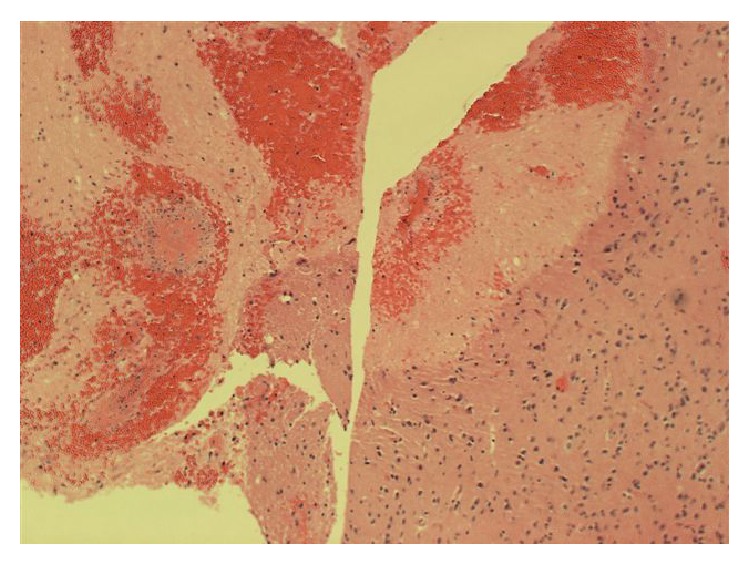
The stark contrast between normal brain tissue to the right (having few inflammatory cells) and the necrotic hemorrhagic tissue to the left.

**Figure 3:**
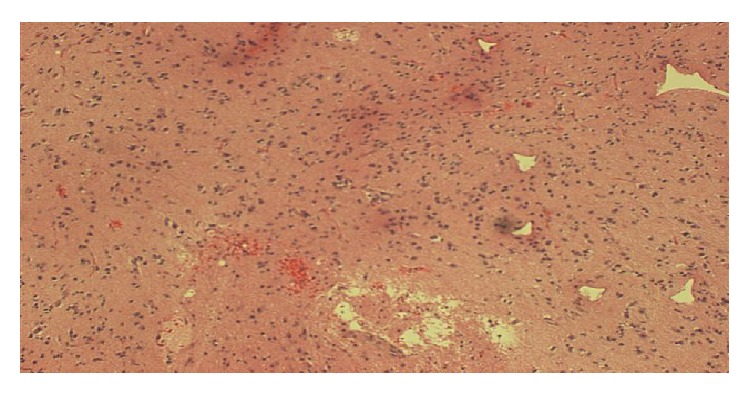
Brain tissue that is beginning to undergo changes of coagulation necrosis with focal hemorrhage and focal necrosis. There is tissue reaction with increased number of astrocytes, neutrophils, and edema.

**Figure 4:**
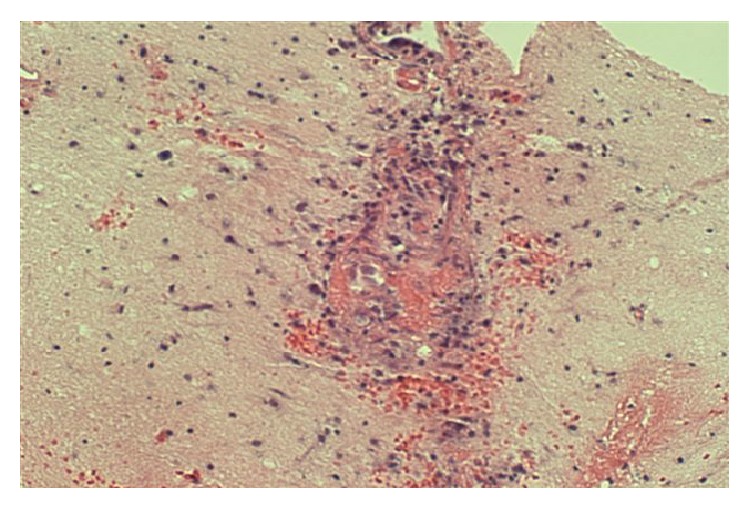
Clear features of vasculitis: a small sized vessel with endothelial cell edema, infiltration by lymphocytes, neutrophils and possibly macrophages, necrosis, and hemorrhage. There is also brain parenchyma necrosis around the affected vessel.

**Figure 5:**
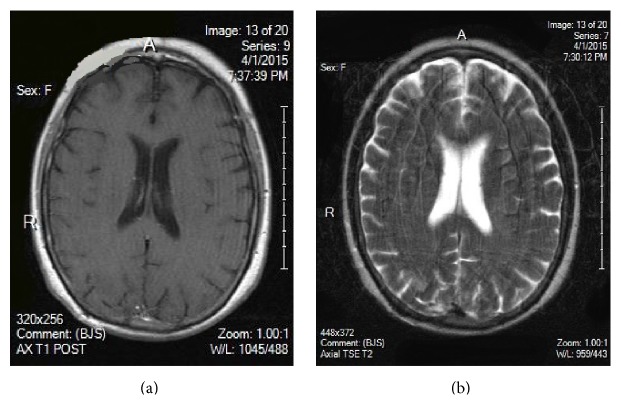
(a), (b) One-month follow-up: no abnormal enhancement; no mass or mass effect. Burr hole for the brain biopsy can be seen in the right posterior lobe.

Over the next 48 hours, an invesitagation to the cause of the abnormal MRI proved to be nondiagnostic. An infectious disease workup was unrevealing and is listed in Table 1(b). A urine and serum toxicology test showed no drug usage. A malignancy workup was performed, with a CT scan of her chest, abdomen, and pelvis showing no evidence of neoplasm. Her abdominal CT scan also showed no active bowel inflammation from UC. During this period, with supportive care, the patient did improve mentally. She became more cooperative and conversant but was still not oriented to time or her age and displayed short- and long-term memory deficits. With the patient's severe impairment, coupled with the negative testing and the mystery of her condition, it was concluded that a brain biopsy would be appropriate, and neurosurgery was consulted. A lumbar puncture was discussed, but ultimately deferred in favor of the brain biopsy. Four days after admission, the patient underwent a stereotactic guided biopsy of the right occipital brain lesions without complication. Pathology revealed coagulation necrosis of parenchyma and vessels. The lesions lacked the selective necrosis of oligodendrocytes and macrophage infiltration of classic demyelinating disease such as multiple sclerosis (MS). Toxoplasma and TB was negative histologically and immunohistochemically. A Giemsa-Wright (GW) stain as well as a Gomori-Grocott methenamine silver (GMS) stain was negative for fungi, parasites, and viral inclusion bodies. No neoplasm was identified. However, there were features of vasculitis on a few biopsy fragments showing small sized vessels with endothelial cell edema, infiltration by lymphocytes, neutrophils, and possibly macrophages, and necrosis and hemorrhage (Figures 2, 3, and 4). At this point, it was determined that the patient's condition was due to central nervous system vasculitis. The rheumatology service was consulted, and the patient was initially given methylprednisolone 125 mg intravenously and then started on 40 mg IV daily. Rheumatology labs ordered were grossly negative and are listed in Table 1(c). A final diagnosis of ulcerative colitis associated central nervous system vasculitis was rendered. The patient was continued on a tapering dose of steroids, and her memory and cognition rapidly improved back to her baseline. She was discharged on oral prednisone 25 mg daily. On one-month follow-up, a repeat brain MRI showed resolution of the lesions, and there was no mass or mass effect and no abnormal enhancement (Figures 5(a) and 5(b)). On the most recent follow-up 4 months after discharge, she continues to do well and has successfully tapered prednisone down to 15 mg daily, with no adverse neurological effects noted. Her IBD continues to be quiescent.

## 3. Discussion

UC associated CNS vasculitis is a rare neurological manifestation of systemic inflammatory bowel disease. Though the exact incidence specifically of CNS vasculitis secondary to UC is not known, it is estimated that cerebrovascular manifestations are seen in 0.12–4% of inflammatory disease patients [5]. These include arterial and venous thrombosis, seizures, leukoencephalitis, and vasculitis [6]. Unnikrishnan et al. report that there have only been fifteen case reports of possible cerebral vasculitis in UC. Among these reported cases, eleven patients had confirmed vasculitis based on histopathology, angiogram, or serology [6]. Four patients out of the 15 had brain biopsies. Three of the patients with brain biopsies had necrotizing vasculitis of small and medium sized arteries on histopathology. This finding was similar to our patient. One of the cases that did not undergo brain biopsy did have T2 hyperintensity of the centrum semiovale, which our patient had. This finding has been reported as typical of microangiopathy associated with CNS vasculitis [6, 7]. Almost all 15 cases had recovery of their neurological deficits with prednisone use, while two patients showed recovery with prednisone combined with cyclophosphamide, and one case recovered with a combination of prednisone, azathioprine, and cyclosporine A. Steroid treatment does seem to afford the best outcome for CNS vasculitis, whether associated with UC or not. However, the optimal length of treatment has not been determined. Salvarani et al. report that, on retrospective analysis of 163 adult patients diagnosed with primary CNS vasculitis (PCNSV) over a 29-year period, 85% of the patients treated with prednisone alone responded favorably. Relapses were observed in 27% of the total patients [8]. Our patient continues to do well on a slow prednisone taper. How long she will remain on prednisone is yet to be determined, and the dose may have to be increased or supplemented with additional immunosuppressant medication should she have a relapse.

Regarding serological markers, ANA, APL, ANCA, PR3, and MPO antibodies are generally negative in PCNSV, as it is a diagnosis based on tissue pathology and neurovascular studies, with no underlying connective tissue or systemic vasculitis disease. However, PR3 ANCA has been noted to occur in UC, with a reported incidence varying from 4 to 43% [6]. PR3 ANCA, the classic serological marker in granulomatosis with polyangiitis (GPA), is known to cause aggressive vasculitis in GPA patients that can rarely involve the brain. In small cohort studies of UC patients, PR3 ANCA has been associated with shorter disease duration, but more extensive colitis, without gender predilection [6, 9]. Unnikrishnan et al. do report a case of PR3 ANCA associated CNS vasculitis in a patient with UC, and of the 15 previously reported cases of CNS vasculitis associated with UC, one MPO antibody mediated systemic vasculitis with multiple brain infarcts was noted. Serological testing is appropriate in patients suspected of CNS vasculitis, so as to exclude underlying connective tissue disease as a cause. Our case is considered vasculitis secondary to a systemic inflammatory condition; in this case, ulcerative colitis and rheumatology serological markers would be assumed to be negative, as they turned out to be. There also does not seem to be a relation between status of the inflammatory bowel disease and the development of CNS vasculitis, and the neurological manifestations may occur independent of the activity of the bowel disease [2, 5, 10]. Indeed, our patient's bowl disease was well controlled with no other systemic manifestations. In regard to pathogenesis of vasculitis in UC, most authors implicate immune mediated mechanisms, possibly via genetic susceptibility and HLA status, T lymphocyte-mediated cytotoxicity, or immune complex deposition [2]. The differential diagnosis of CNS vasculitis with brain lesions can be varied and complex. Careful exclusion of other disorders is essential. The major categories of differential diagnosis to consider include PRES, reversible cerebral vasoconstriction syndrome (RCVS), connective tissue disease, infections, embolic disease, atherosclerosis, systemic vasculitis, malignancy, cocaine induced vasculitis, and other white matter diseases. PRES was initially considered in this patient due to the occipitoparietal edema on MRI, which is characteristic of this condition. PRES is most often associated with uncontrolled hypertension, eclampsia, cancer chemotherapy, and bone marrow or stem cell transplantation. Aside from high blood pressure, the patient did not have any of the other conditions. PRES patients typically exhibit complete neurological recovery within days when the underlying condition is treated. In our patient, the brain lesions and AMS continued to be present even when her blood pressure normalized. In addition, PRES brain biopsies show vasogenic edema and scattered lymphocytes without inflammation, ischemia, or neuronal damage [11, 12].

A consideration should be made to demyelinating disorders such as multiple sclerosis (MS), which are noted to have an increased prevalence in patients with IBD. In a large retrospective study, it was noted that 0.5% of CD patients had also been diagnosed with MS, while the estimated prevalence of MS in the general population is only about 0.1%. Other studies have also highlighted an increased risk of MS in IBD patients, as compared with a matched control group [1, 13]. Another potential concern to consider is the IBD patient with sudden neurological symptoms who has recently been treated with biologics such as tumor necrosis factor alpha inhibitor (anti-TNF) drugs. These medications have been reported to trigger CNS white matter lesions as a rare side effect [1, 14]. This has led to some dispute whether the use of biologics and the development of white matter lesions are real or are explained by the underlying disease for what the medication is used for. Alternatively, demyelinating lesions have also been reported in patients taking anti-TNFs for other diseases such as rheumatoid arthritis and so the current thinking is that both the underlying disease and treatments may play a role [1, 14]. In addition, the resulting immunosuppression afforded by anti-TNFs may lead to opportunistic infections causing white matter disease such as JC virus mediated progressive multifocal leukoencephalopathy. Our patient had never been on anti-TNF medication. A thorough history and physical coupled with appropriate serological markers and investigations for infection and malignancy are warranted. Imaging studies, lumbar puncture, and cerebral angiograms can be vital in differentiating between the various causes of brain lesions. And, as was done in our patient, brain biopsy may be necessary to ultimately diagnose the patient's condition.

## 4. Conclusion

Our case of CNS vasculitis associated with UC represents a rare manifestation of IBD, with only a handful of reported cases in the literature. This association should be considered in any patient with IBD who presents with neurological symptoms. While the differential diagnosis is extensive and necessitates a well-constructed workup, a brain biopsy may ultimately be necessary to diagnose this condition. The neurological manifestations and gross abnormalities on MRI may be disconcerting, but the majority of these patients do respond well to steroid treatment, as evidenced by the dramatic resolution of the brain lesions seen in our patient. However, the length of treatment has not been firmly established. Hopefully, with more documentation of this rare association, better diagnostic and treatment parameters may be established.

## Figures and Tables

**(a):** 

Lab	Results	Normal
CRP	6.31 mg/dL	<.08 mg/dL
Hemoglobin	12.6 g/dL	11.7–15.5 g/dL
ESR	19 mm/h	<30
White blood count	15.9	3.4–10.8
Creatine kinase level	57 u/L	29–143
CMP	Normal	Normal
EKG	Normal sinus rhythm	Normal
Urine analysis	No protein No red blood cells	0 mg/dL 0–2/hpf

**(b):** 

Lab	Result
HIV	Negative
HSV Ab	Negative
TB by IGRA	Negative
Toxoplasmosis Ab	Negative
Blood cultures	Negative
Urine toxicology	No drug usage
Serum toxicology	No drug usage

**(c):** 

Lab	Result
ANA	Negative
SSA	Negative
SSB	Negative
HLA B27	Negative
P ANCA	Negative
C ANCA	Negative
Anti-MPO	Negative
APL	Negative
RF	Negative
CCP	Negative
